# Twin-Core Fiber-Based Mach Zehnder Interferometer for Simultaneous Measurement of Strain and Temperature

**DOI:** 10.3390/s18030915

**Published:** 2018-03-20

**Authors:** Dominik Kowal, Waclaw Urbanczyk, Pawel Mergo

**Affiliations:** 1 Department of Optics and Photonics, Faculty of Fundamental Problems of Technology, Wroclaw University of Science and Technology, 50-370 Wroclaw, Poland; waclaw.urbanczyk@pwr.edu.pl; 2 Laboratory of Optical Fiber Technology, Maria Curie-Sklodowska University, 20-031 Lublin, Poland; pawel.mergo@poczta.umcs.lublin.pl

**Keywords:** fiber-optic sensors, twin-core fibers, simultaneous measurement of strain and temperature

## Abstract

In this paper we present an all-fiber interferometric sensor for the simultaneous measurement of strain and temperature. It is composed of a specially fabricated twin-core fiber spliced between two pieces of a single-mode fiber. Due to the refractive index difference between the two cores in a twin-core fiber, a differential interference pattern is produced at the sensor output. The phase response of the interferometer to strain and temperature is measured in the 850–1250 nm spectral range, showing zero sensitivity to strain at 1000 nm. Due to the significant difference in sensitivities to both parameters, our interferometer is suitable for two-parameter sensing. The simultaneous response of the interferometer to strain and temperature was studied using the two-wavelength interrogation method and a novel approach based on the spectral fitting of the differential phase response. As the latter technique uses all the gathered spectral information, it is more reliable and yields the results with better accuracy.

## 1. Introduction

Fiber-optic interferometers have been widely used for measurement applications [1] because of their small sizes, possibility of remote operation, high measurement precision, and sensitivity. In the classical configuration, two fibers are used as interferometer arms, which requires fiber-optic couplers for splitting the light and recombining it at the interferometer output. Much better compactness can be achieved in so-called inline fiber interferometers, which integrate two arms in a single piece of fiber. In such a case, the splitting and recombining of the interfering beams are accomplished by tapering the fiber [2], splicing it to a different kind of fiber [3], or using fiber gratings [4]. The inline architecture allows for miniaturization of the sensing head and reduces the sensor cost. Many examples of using inline interferometers for measuring hydrostatic pressure [5], strain [6], temperature [7,8], bending [9], or refractive index [10] can be found in the literature. Among the others, the twin-core fibers (TCFs) are very promising for applications in inline interferometers. They offer a range of possibilities, particularly the independent tailoring of the sensing characteristics of both arms by core geometry and level of doping. To date, TCFs have been employed in interferometric devices devoted to measuring strain [11], temperature [12], or refractive index [13].

Overcoming the cross-sensitivity to temperature when measuring other physical parameters is one of the challenges in fiber-optic sensing. In this paper, we address the issue of the simultaneous measurement of strain and temperature with the use of an interferometric sensor based on a specially designed TCF. Several techniques for achieving this goal have been already presented. These include the use of fiber grating sensors [14], fluorescence and Brillouin scattering in erbium-doped fibers [15], and interferometric techniques [16,17,18]. In [16], a Mach–Zehnder interferometer (MZI), formed by a thin core fiber spliced between two sections of single-mode fiber (SMF), was used for the simultaneous measurement of strain and temperature by analyzing both the shift of fringe pattern and change in fringe visibility. A different type of MZI was proposed in [17], employing a double-cladding fiber and two long-period gratings. In such a case, the interference fringe and spectral envelope shifts were measured under strain and temperature variations. Finally, in [18], Frazao et al. showed the possibility of the simultaneous measurement of strain and temperature using a hybrid Fabry–Perot/Michelson reflection-type interferometer based on a microstructured TCF. In such a device, the shifts of fringe patterns produced by the Michelson and Fabry–Perot interferometers were monitored separately to recover information about the two parameters. To the best of our knowledge, this was the first time that a TCF was employed in a sensor capable of discriminating the responses to strain and temperature. A certain drawback of this solution is related to the fact that the twin-core microstructured fiber used as a sensing element is more difficult in fabrication and in making resistant splices than the conventional TCFs. In this paper, we propose a simpler approach, based on a single inline MZI employing a conventional TCF with zero-sensitivity to elongation at certain wavelengths and spectral interrogation of the interference signal. In contrast to [16,17,18], we only analyze the shift of one interference pattern created by the modes propagating in the two cores. The device shows a significant difference in the spectral response to temperature and strain, thus providing an opportunity for the simultaneous measurement of the two parameters. Both sensitivities can be tuned simply by varying the length of the TCF. From the simultaneous differential phase response of the interferometer to temperature and strain measured vs wavelength, the value of the two parameters is retrieved by the use of two alternative methods. First, we show the results obtained by a commonly used matrix method. This was supported by the careful choice of the interrogation wavelengths to obtain the highest-possible determinant of the sensitivity matrix. We have also proposed a novel retrieval method based on fitting the simultaneous response to temperature and strain changes with spectral sensitivity curves, measured individually for each parameter. We demonstrate that such an approach significantly improves the resolution of simultaneous measurements compared to the traditional two-wavelength interrogation approach. Another advantage of this technique is in enlarging the free spectral range (FSR) of the sensor and therefore extending the measurement range.

## 2. Interferometer Design

**Figure 1:**
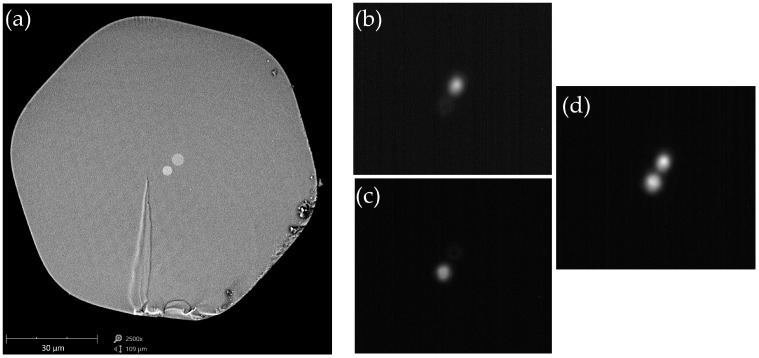
(**a**) Cross-section of the twin-core fiber (TCF) and pictures of mode fields where (**b**) the smaller core is excited; (**c**) the larger core is excited; and (**d**) both cores are equally excited.

The TCF with the cross-section presented in Figure 1a was fabricated in the Laboratory of Optical Fiber Technology, Maria Curie-Sklodowska University, Lublin, Poland. The fiber had two cores with different levels of germanium doping and different diameters. A smaller core (3 μm in diameter, doping level of 6 mol %) is located in the cladding center, whereas a larger one (4 μm in diameter, doping level of 3.5 mol %) is shifted by 5 μm off the symmetry axes of the cladding. The refractive index difference between the cores is 3 × 10^−3^. The outer diameter of the fiber was equal to 100 μm. Both cores were designed to have approximately the same cutoff wavelength of about 800 nm. Thus, in the observed range of 800–1300 nm, only the fundamental modes were supported in both cores. Although the cores were relatively close to each other, a large refractive-index contrast ensured that there was no significant mode coupling in the straightened fiber at a short wavelength range. This statement is supported by the observation of mode fields of the TCF for different excitation conditions at *λ* = 800 nm (Figure 1b–d). In this experiment, the light was coupled to the TCF from Thorlabs SM600 fiber (Thorlabs, Newton, NJ, USA). By proper alignment of the leading-in fiber, we were able to individually excite each core with the power crosstalk to the adjacent core lower than 9%. It is a significant advantage of our TCF that the two cores can be treated independently at a short wavelength range, which allows individual tuning of their responses to the measured physical parameters by adjusting the doping level and diameter. This is not the case in intermodal inline MZIs [5], where the interference occurs between the fundamental and higher-order mode. In such a case, the difference in the response of the two modes can only arise due to different confinement coefficients. 

**Figure 2:**
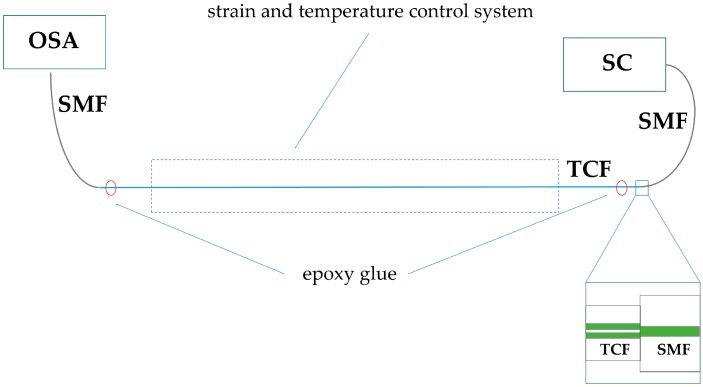
Experimental setup for measuring the elongation and temperature response of the SMF–TCF–SMF interferometer. SC: supercontinuum; SMF: single mode fiber; TCF: twin core fiber; OSA: optical spectrum analyzer. On the inset, the splice between the SMF and TCF is shown schematically. Cores in both fibers are indicated by green color.

**Figure 3:**
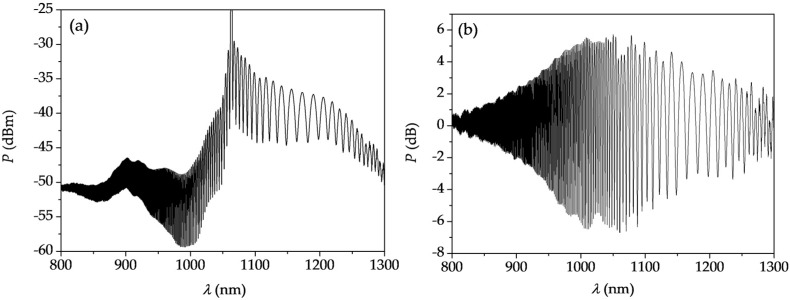
(**a**) Spectrum of light transmitted through the SMF–TCF–SMF interferometer; and (**b**) transmission spectrum normalized to the source characteristics.

The sensor design is schematically shown in Figure 2. The TCF of length *L* = 36 cm was spliced with the two pieces of a single-mode fiber (SMF). The first splice was made after the precise lateral alignment of the two fibers to ensure equal power coupling between the SMF and the twin cores. The lateral alignment at the second splice was optimized for the maximum contrast of the spectral interference fringes observed at the output of the SMF (see Figure 3a). The three pieces of the fibers composed an inline MZI, which was examined for elongation and temperature response. For this purpose, we used broad-band light from the supercontinuum source (SC) NKT Photonics SuperK (NKT Photonics, Birkerød, Denmark) coupled to the first SMF, and the transmission spectrum was acquired with the Optical Spectrum Analyzer (OSA) Yokogawa AQ6370D (Yokogawa Electric Corporation, Musashino, Tokyo, Japan) at the output of the second SMF, with the resolution set to 0.2 nm. The TCF was attached to a fixed stage and a translational stage with epoxy glue. Gluing points were 29 cm apart from each other, and between them, a length of 22 cm of the fiber was put in the temperature controlling system. It was a thin copper pipe surrounded by mass of water with an immersion heater. In this way we could expose the TCF to simultaneous changes in temperature and strain. The specified length of the TCF, *L* = 36 cm, was limited by the size of the temperature controlling system.

Due to a group index difference between the modes propagating in the two cores of the TCF, the group delay is accumulated along the fiber length, and in consequence, the interference pattern is observed in the spectrum of transmitted optical power *P*. Figure 3a shows the spectral interference pattern at the output of the second SMF, after optimizing the lateral alignment between the SMF and TCF for the maximum fringe contrast. In Figure 3b, the transmission spectrum of the TCF is normalized to the source characteristics. The difference in the group refractive indices, Δ*N*, of the modes propagating in the two cores varies between 3 × 10^−3^ at *λ* = 800 nm and 2 × 10^−4^ at *λ* = 1126 nm. These values can be easily obtained from the TCF transmission spectrum using the following relationship:

(1) $$\Delta N=\frac{\lambda_{0}{}^{2}}{L\times \Delta \lambda }$$

where *λ*_0_ is the mean wavelength between two neighboring interference fringes, Δ*λ* is the spectral separation of the neighboring fringes, and *L* is the fiber length.

## 3. Strain and Temperature Sensitivity

**Figure 4:**
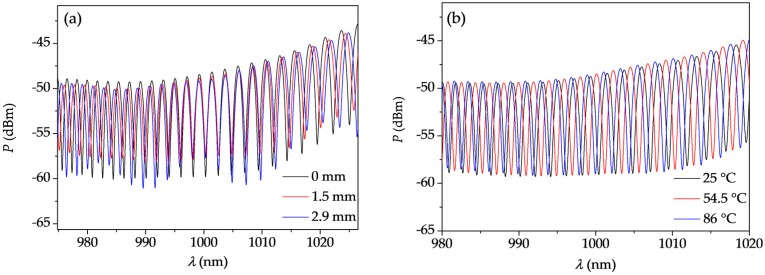
(**a**) Shift of the interference fringes around zero-sensitivity wavelengths upon TCF elongation; and (**b**) shift of the interference fringes upon temperature rise, shown in the same spectral range.

The interferometer was elongated at room temperature (23 °C) in the range of up to 2.90 mm, so the maximum value of elongation corresponds to 10 mε. The translational stage was moved by the micrometer screw with 10 μm resolution (34.5 με). Stretching the fiber resulted in the shift of the interference fringes in different directions, depending on their spectral position. The fringes located in the spectral range below 1000 nm moved towards shorter wavelengths, while the fringes located in the long wavelength range (above 1000 nm) moved in the opposite direction. This effect is shown in Figure 4a, and directly confirms that in the short wavelength range, the fiber elongation results in a differential phase decrease; while in the long wavelength range, the response of the TCF has the opposite sign.

As the differential phase shift *Φ* must fulfil the following condition at each interference minimum:

(2) Φ=2π(m+1/2)

where *m* is a fringe order, one can reconstruct the spectral dependence of the *Φ*(*λ*) (with the precision up to a constant value), if the spectral locations of the interference minima are known. If such a phase reconstruction procedure is conducted for each value of applied strain or temperature, the spectral dependence of the sensitivity for a given parameter may be determined in the following way:

(3) $$K_{X}\left(\lambda \right)=\frac{\Phi (\lambda ,X_{2})-\Phi (\lambda ,X_{1})}{X_{2}-X_{1}}$$

where *X*_1_ and *X*_2_ stand for the initial and final values of the measurement (elongation or temperature).

**Figure 5:**
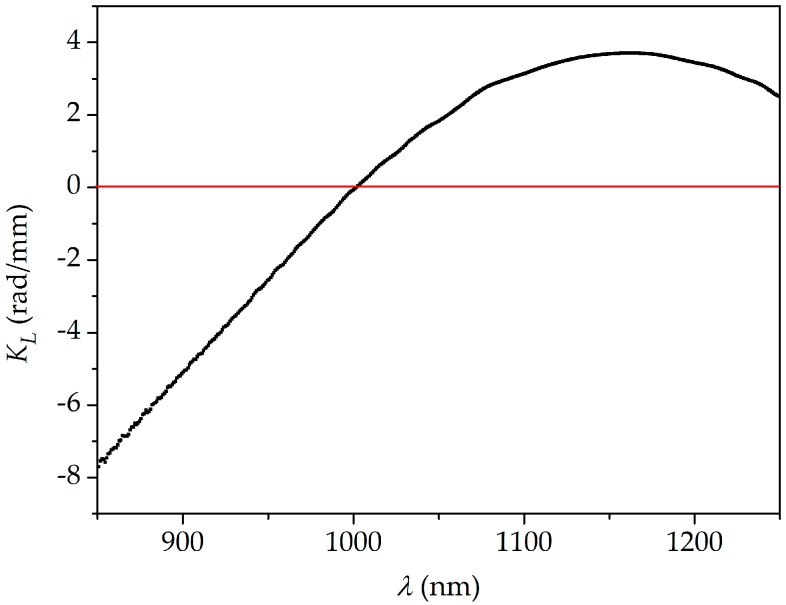
Spectral sensitivity to elongation *K_L_* (black points) measured for the TCF elongation of 2.9 mm (10 mε). The red line indicates zero level.

**Figure 6:**
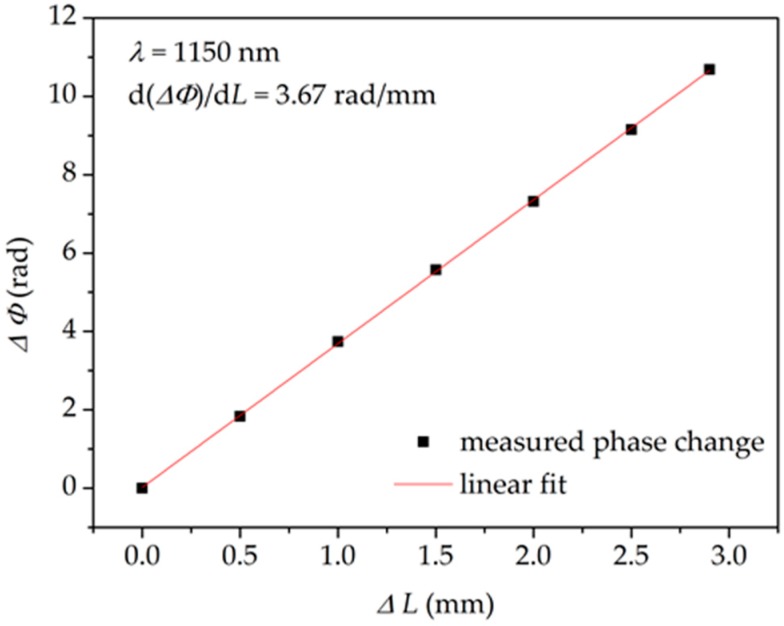
Differential phase shift increase Δ*Φ* of the interferometer induced by fiber elongation measured at *λ* = 1150 nm. ∆*L* = change in length of interferometer.

To determine the spectral sensitivity *K_X_*(*λ*), the spectral range of interest was narrowed to the 850–1250 nm range, as the interference fringes outside these border wavelengths were too noisy. The calculated sensitivity of the interferometer to elongation *K_L_* is shown in Figure 5. This result was obtained for the maximum fiber elongation Δ*L* = 2.9 mm, but the differential phase shift increase Δ*Φ* exhibited a linear response to fiber elongation in the whole spectral range. This is illustrated in Figure 6 for the wavelength of 1150 nm. In this figure, the error bars are not visible as the estimated inaccuracy of Δ*Φ* measurement was ±0.02 radian (rad). This figure represents the root mean square (RMS) deviation between the measured profile of *Φ*(*λ*) and the fitted curve.

**Figure 7:**
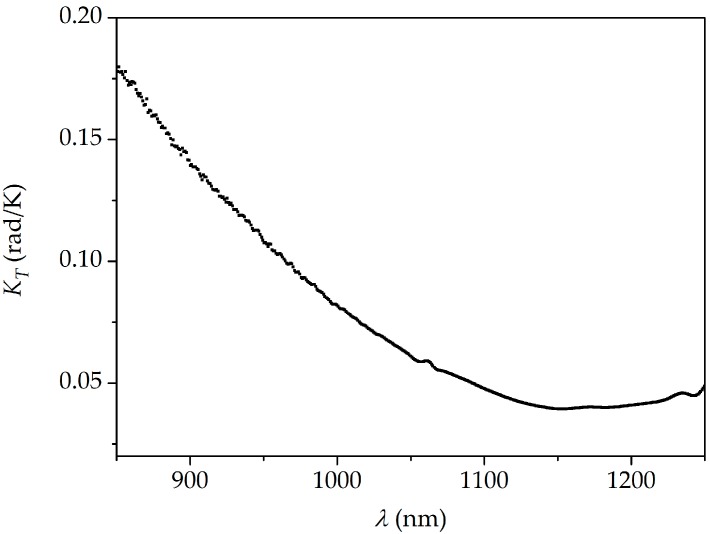
Spectral sensitivity to temperature *K_T_* of the TCF measured for the temperature change from 25 °C to 86 °C.

**Figure 8:**
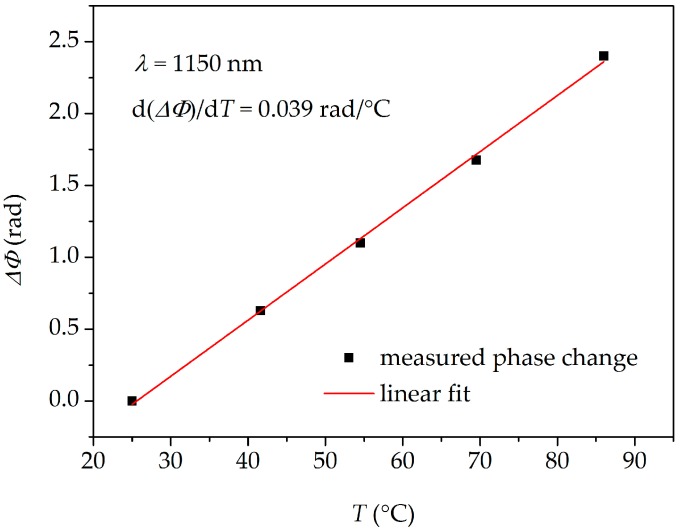
Differential phase shift increase Δ*Φ* of the interferometer induced by temperature (*T*) change at *λ* = 1150 nm.

In the next step, the interferometer was subjected to increasing and decreasing temperature *T* in the range from *T* = 25 °C to *T* = 86 °C. The temperature was measured by a thermocouple with 0.1 °C accuracy. The fiber elongation was kept constant at Δ*L* = 0 during the temperature measurements. The resulting fringe shift was positive (fringes moved towards longer wavelengths) in the whole analyzed spectral range and no hysteresis was observed after cooling the fiber down. The interference fringes registered for three different temperatures are shown in Figure 4b in the 980–1020 nm spectral range. The spectral dependence of the sensitivity to temperature *K_T_* presented in Figure 7 was calculated by subtracting the differential phase shift reconstructed for the highest temperature (86 °C) and the room temperature (25 °C). As it is shown in Figure 8, the response of the TCF to temperature is linear in the investigated temperature range. In this case, similarly as in Figure 6, the Δ*Φ* measurement inaccuracy is ±0.02 rad.

## 4. Simultaneous Measurements of Strain and Temperature

**Figure 9:**
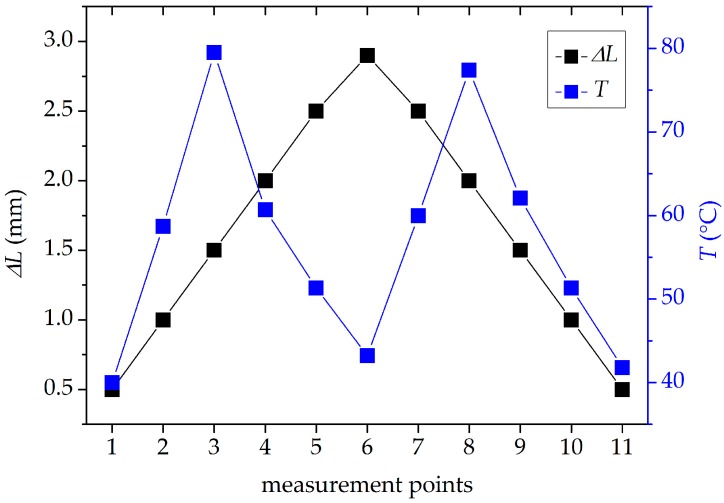
Fiber elongation and temperature values chosen to examine interferometer response to simultaneous changes of the two parameters.

Finally, we tested the performance of the interferometer based on the TCF in the simultaneous measurements of temperature and elongation. The TCF was elongated up to Δ*L* = 2.9 mm (10 mε) by the micrometer screw and loosened, while the temperature was elevated and dropped alternately. The exact elongation and temperature values applied to the TCF in these experiments are depicted by the points in Figure 9, which were chosen in such a way to ensure high diversity in the measurement conditions. The straight lines connecting the points in this figure only serve to improve its clarity. The measured spectral sensitivities of the interferometer to elongation and temperature can be used for retrieving the values of the two parameters from the overall sensor response. This is usually achieved by taking the sensitivities to both parameters at two different wavelengths, namely *K_L_*(*λ*_1_), *K_T_*(*λ*_1_), *K_L_*(*λ*_2_), and *K_T_*(*λ*_2_), and calculating the unknown values of Δ*T* (temperature change in reference to the initial value) and Δ*L* using the following equation:

(4) $$\left[\begin{array}{c} \Delta T\\ \Delta L \end{array}\right]=\frac{1}{D}\;\left[\begin{array}{cc} K_{T}\left(\lambda_{2}\right) & -K_{T}\left(\lambda_{1}\right)\\ -K_{L}\left(\lambda_{2}\right) & K_{L}\left(\lambda_{1}\right) \end{array}\right]\;\left[\begin{array}{c} \Delta \Phi \left(\lambda_{1}\right)\\ \Delta \Phi \left(\lambda_{2}\right) \end{array}\right]\;$$

where *D* = *K_L_*(*λ*_1_) × *K_T_*(*λ*_2_) − *K_T_*(*λ*_1_) × *K_L_*(*λ*_2_) is a determinant of the sensitivity matrix and Δ*Φ* is a differential phase shift increase of the interferometer, induced by the simultaneous change in temperature and elongation.

**Figure 10:**
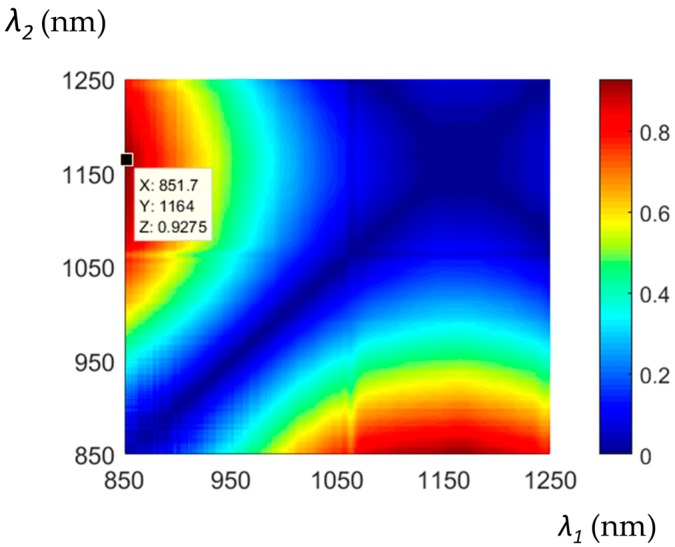
Absolute value of the sensitivity matrix determinant *D* for different wavelength pairs. Data cursor shows the optimized choice of wavelengths.

For the precise determination of Δ*T* and Δ*L*, it is required that the absolute value of the determinant *D* is the highest possible, otherwise inaccuracy in the Δ*Φ* measurement is enlarged in the reconstructed parameters of Δ*T* and Δ*L*. Therefore, we have calculated the determinant *D* over the whole spectrum and resulting values are shown as a two-dimensional map in Figure 10. In such a way, we were able to select the best wavelengths for the recovery of the two parameters, which are *λ*_1_ = 851.7 nm and *λ*_2_ = 1164 nm. The free spectral range (FSR) of our sensor at these two wavelengths is equal to FSR (*λ*_1_) = 0.66 nm and FSR (*λ*_2_) = 17 nm, respectively. The shift of the interference fringes exceeding these values loses unambiguity. Therefore, the maximum elongation and temperature increase, which can be measured by this method without ambiguity, are equal to Δ*L* = 0.84 mm and *T* = 61 °C, respectively. These numbers are only applicable when elongation and temperature are measured separately. When simultaneous measurement is performed, the fringe shift is a sum of temperature- and elongation-induced shifts. In order to measure large changes in Δ*T* and Δ*L*, one must take care to register the interference spectra at subsequent measurement points corresponding to a phase shift increase of lower than 2*π*. 

**Figure 11:**
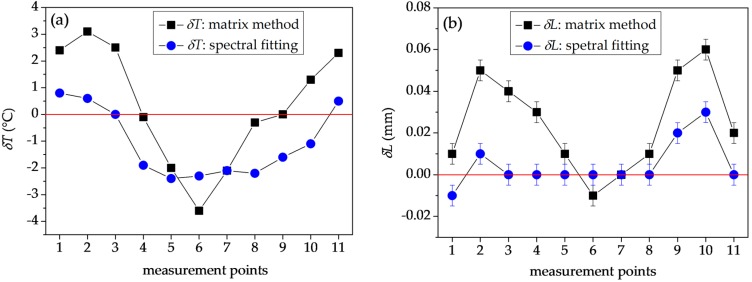
The differences between applied values of (**a**) temperature and (**b**) elongation, and the values retrieved by the two-wavelength method and spectral fitting method. The red lines indicate zero levels.

The differences between the retrieved and applied values of temperature and elongation *δT* and *δL* are shown in Figure 11a,b. Similarly as in Figure 9, the straight lines are drawn between the data points to improve clarity of the figure. The error bars in Figure 11a are not visible as they are smaller than the size of the data points (0.1 °C). To estimate the accuracy of this technique, we calculated the RMS deviations between the measured and applied values of the two parameters, which are respectively equal to *σ_T_* = 2.13 °C and *σ_L_* = 0.033 mm (*σ_ε_* = 114 με). As the determinant of the sensitivity matrix takes the maximum value for the used combination of wavelengths, it is not expected that any other pair of wavelengths could yield the results with better accuracy.

**Figure 12:**
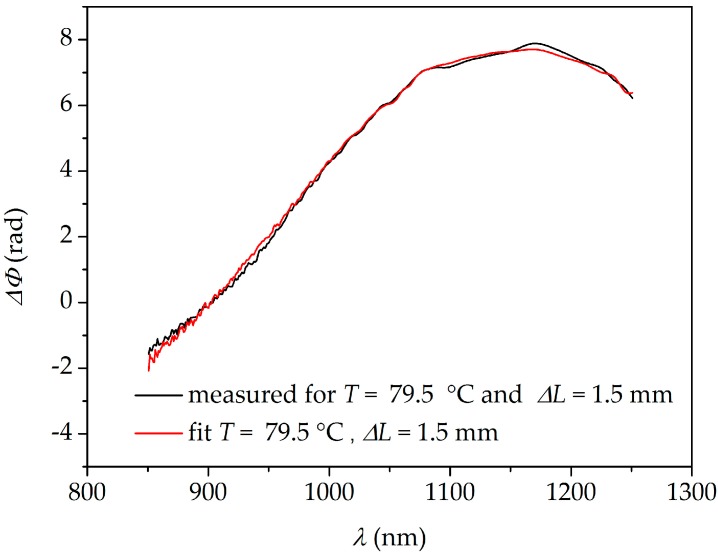
Measured differential phase shift response of the interferometer to simultaneously applied elongation and temperature, and the best fit thereof.

We have also applied a different method of retrieving the two parameters, taking advantage of the broad-band spectral response of the interferometer and therefore providing much better accuracy than the two-wavelength interrogation. This approach is based on a spectral fitting of the overall differential phase response Δ*Φ*(*λ*), with a sum of *K_T_*(*λ*) and *K_L_*(*λ*) functions with Δ*T* and Δ*L* weight coefficients. The optimization of the fit is conducted using MATLAB R2016a software package. The Euclidean norm of the difference between experimental and fitted data is calculated and it is minimized using the Nelder–Mead algorithm. The resulting fit yields the results for Δ*T* and Δ*L*. It is intuitively understandable that this technique is more reliable than the two-wavelength method, as it uses the information from the whole interference spectrum. Therefore, the negative influence of measurement noise is greatly reduced. The example of the mixed interferometer response and the fitting outcome is shown in Figure 12. In Figure 11a,b, we show the differences between the retrieved and applied values of Δ*T* and Δ*L* obtained by the spectral fitting method. In this case, the RMS deviations from the measured values of Δ*T* and Δ*L* are *σ_T_* = 1.62 °C and *σ_L_* = 0.012 mm (*σ_ε_* = 41 με). This is a significant improvement as compared to the results obtained by the two-wavelength interrogation method. Further increases in accuracy could be achieved by making a TCF interferometer longer, as the same strain and temperature changes applied to a longer TCF will result in a greater differential phase shift increase Δ*Φ*. Furthermore, the unambiguous measurement range limited by the FSR in case of the two-wavelength interrogation method is greatly increased for the spectral fitting method. As all the spectral data are processed simultaneously, the wavelength corresponding to zero-sensitivity to elongation serves as a reference point and allows for practically unlimited elongation measurements. This reference fringe is obviously affected by temperature when the two parameters are changed simultaneously, and it will experience the 2*π* differential phase increase with a temperature change of 103.5 °C. Therefore, this value determines the unambiguous measurement range for temperature. A certain drawback of the spectral fitting method is that it takes more time to perform the fitting than to solve a set of two linear equations.

## 5. Conclusions

In this paper, we studied the performance of the inline interferometric sensor for temperature and elongation measurements based on a specially designed TCF. The differential phase response of the TCF was measured in the 850–1250 nm spectral range, showing that there is one wavelength insensitive to fiber elongation, whereas the temperature sensitivity is nonzero in the whole analyzed spectral range. Such sensing characteristics of the TCF allowed for the simultaneous measurements of elongation and temperature. Firstly, the two-wavelength interrogation method was employed for this purpose, and it yielded the results with the RMS deviations of 2.13 °C and 0.033 mm (114 με). We also proposed to use a spectral fitting technique providing the improvement in the RMS deviation up to 1.62 °C and 0.012 mm (41 με). Although the spectral fitting takes more time to retrieve the applied values of temperature and elongation, this technique uses all the gathered spectral information, and therefore is less sensitive to measurement noise and yields more accurate results. Also, the spectral fitting method provides a much greater unambiguous measurement range. An additional advantage of our sensor is simplicity, allowing for its various practical applications. In terms of measurement resolution, it shows a similar performance to grating-based sensors [14] assuring a resolution in two-parameter measurements equal to 1.2 °C and 25.8 με, respectively. Moreover, the sensitivity of our device can be easily tuned, simply by increasing the length of the TCF.

## References

[1] Lee B.H., Kim Y.H., Park K.S., Eom J.B., Kim M.J., Rho B.S., Choi H.Y. (2012). Interferometric fiber optic sensors. Sensors.

[2] Tian Z., Yam S.S.-H., Barnes J., Bock W., Greig P., Fraser J.M., Loock H.-P., Oleschuk R.D. (2008). Refractive Index Sensing With Mach–Zehnder Interferometer Based on Concatenating Two Single-Mode Fiber Tapers. IEEE Photonics Technol. Lett..

[3] Kim B., Kim T.-H., Cui L., Chung Y. (2009). Twin core photonic crystal fiber for in-line Mach-Zehnder interferometric sensing applications. Opt. Express.

[4] Lim J.H., Jang H.S., Lee K.S., Kim J.C., Lee B.H. (2004). Mach–Zehnder interferometer formed in a photonic crystal fiber based on a pair of long-period fiber gratings. Opt. Lett..

[5] Statkiewicz-Barabach G., Olszewski J., Mergo P., Urbanczyk W. (2017). Hydrostatic Pressure and Temperature Measurements Using an In-Line Mach-Zehnder Interferometer Based on a Two-Mode Highly Birefringent Microstructured Fiber. Sensors.

[6] Zhang C., Ning T., Li J., Zheng J., Gao X., Pei L. (2012). Refractive index and strain sensor based on twin-core fiber with a novel T-shaped taper. Opt. Laser Technol..

[7] Nguyen L.V., Warren-Smith S.C., Ebendorff-Heidepriem H., Monro T.M. (2016). Interferometric high temperature sensor using suspended-core optical fibers. Opt. Express.

[8] Li L., Xia L., Xie Z., Hao L., Shuai B., Liu D. (2012). In-line fiber Mach–Zehnder interferometer for simultaneous measurement of refractive index and temperature based on thinned fiber. Sens. Actuators A Phys..

[9] Zhang S., Zhang W., Gao S., Geng P., Xue X. (2012). Fiber-optic bending vector sensor based on Mach–Zehnder interferometer exploiting lateral-offset and up-taper. Opt. Lett..

[10] Tian Z., Yam S.S.-H. (2009). In-Line Single-Mode Optical Fiber Interferometric Refractive Index Sensors. J. Lightwave Technol..

[11] Feng S., Li H., Xu O., Lu S., Jian S. Compact in-fiber Mach-Zehnder interferometer using a twin-core fiber. Proceedings of the 2009 Asia Communications and Photonics Conference and Exhibition (ACP).

[12] Rugeland P., Margulis W. (2012). Revisiting twin-core fiber sensors for high-temperature measurements. Appl. Opt..

[13] Zhou A., Li G., Zhang Y., Wang Y., Guan C., Yang J., Yuan L. (2011). Asymmetrical Twin-Core Fiber Based Michelson Interferometer for Refractive Index Sensing. J. Lightwave Technol..

[14] Zhou D.P., Wei L., Liu W.K., Lit J.W.Y. (2009). Simultaneous Strain and Temperature Measurement with Fiber Bragg Grating and Multimode Fibers Using an Intensity-Based Interrogation Method. IEEE Photonics Technol. Lett..

[15] Ding M., Mizuno Y., Nakamura K. (2014). Discriminative strain and temperature measurement using Brillouin scattering and fluorescence in erbium-doped optical fiber. Opt. Express.

[16] Zhou J., Liao C., Wang Y., Yin G., Zhong X., Yang K., Sun B., Wang G., Li Z. (2014). Simultaneous measurement of strain and temperature by employing fiber Mach-Zehnder interferometer. Opt. Express.

[17] Kim M.J., Kim Y.H., Lee B.H. (2008). Simultaneous measurement of temperature and strain based on double cladding fiber interferometer assisted by fiber grating pair. IEEE Photonics Technol. Lett..

[18] Frazão O., Silva S.F., Viegas J., Baptista J.M., Santos J.L., Roy P. (2010). A hybrid Fabry–Perot/Michelson interferometer sensor using a dual asymmetric core microstructured fiber. Meas. Sci. Technol..

